# Management of primary hepatic pregnancy: A case report

**DOI:** 10.1016/j.crwh.2024.e00668

**Published:** 2024-11-18

**Authors:** Yusuf Mohammed Yusuf, Gulilat Tigiye Endeshaw, Berhanu Mohammed Shifa, Biniyam Afework Abate, Ashenafi Aberra Buser, Mohammednur Ali Mohammed, Shimelis Ayalew Yimer, Yabets Tesfaye Kebede, Bekri Delil Mohammed

**Affiliations:** a Department of Surgery, Ethio-Tebib General Hospital, Addis Ababa, Ethiopia; b Department of Surgery, St Paul's Hospital Millennium Medical College, Addis Ababa, Ethiopia; c Department of Obstetrics and Gynecology, Ethio-Tebib General Hospital, Addis Ababa, Ethiopia; d Department of Radiology and Medical Radiology Technology, St Paul's Hospital Millennium Medical College, Addis Ababa, Ethiopia; e Department of Radiology, Ethio-Tebib General Hospital, Addis Ababa, Ethiopia; f Department of Pathology, Ethio-Tebib General Hospital, Addis Ababa, Ethiopia; g Faculty of Medicine, Institute of Health, Jimma University, Jimma, Ethiopia; h Department of Internal Medicine, Ethio-Tebib General Hospital, Addis Ababa, Ethiopia

**Keywords:** Primary hepatic pregnancy, Ectopic pregnancy, Liver resection

## Abstract

Hepatic pregnancy, an exceedingly rare subtype of abdominal ectopic pregnancy, remains clinically challenging due to its infrequency, diverse presentations, and diagnostic difficulties. We report the clinical course, diagnostic journey and treatment of a woman with a primary hepatic pregnancy.

The patient presented with acute pain in the right hypochondrium and vomiting. Imaging revealed a peripheral hypodense gestational sac within the right hepatic lobe containing a fetus with heart pulsations, as well as peritoneal fluid and pelvic collection. Following administration of mifepristone, the patient underwent a laparotomy, and expelled a developed fetus. Hepatic resection utilizing the Pringle maneuver was performed, and methotrexate was administered postoperatively. The patient had a stable recovery and vital signs and was discharged two days after surgery.

This case highlights the diagnostic and management challenges of hepatic pregnancy, and emphasizes the need for heightened clinical suspicion and thorough evaluation. By sharing the experience, we aim to contribute insights to guide the diagnosis and management of similar cases.

## Highlights

• Abdominal ectopic pregnancies may present with gastrointestinal symptoms, leading to misdiagnosis.
• High suspicion and timely imaging are vital to avoid life-threatening complications.
• Transvaginal ultrasound may miss abdominal ectopic pregnancies.
• Preoperative mifepristone, hepatic resection, and methotrexate were used to treat this case.

## 1 Introduction

Ectopic pregnancy, defined as the implantation of a fertilized ovum outside the uterine cavity, is a potentially life-threatening condition that requires prompt diagnosis and management. It often occurs in the fallopian tubes. Abdominal pregnancies, particularly in the liver, are a rare and challenging clinical entity. Hepatic ectopic pregnancies constitute only 0.03 % of all ectopic pregnancies [1].

Primary hepatic pregnancy refers to the implantation of the gestational sac directly on the liver surface. This condition poses significant diagnostic and therapeutic challenges due to its atypical clinical presentation, which often mimics other gastrointestinal or hepatobiliary disorders, leading to delays in recognition and management [2]. Moreover, the potential for life-threatening hemorrhage and the intricacy of surgical interventions contribute to the elevated maternal mortality rate associated with hepatic pregnancies [3].

This case report describes the first documented case in Ethiopia in the literature to our knowledge. It emphasizes the imperative for healthcare professionals to maintain a broad differential diagnosis and exercise heightened vigilance when encountering unusual clinical scenarios. By delineating the diagnostic and management challenges faced in this unique case, the report provides a valuable resource for healthcare practitioners, offering insights into the nuanced approaches required for the effective management of hepatic pregnancies. This contribution enhances the collective understanding of this rare phenomenon, ultimately guiding clinical practice and optimizing patient care.

## 2 Case Presentation

A 35-year-old woman, gravida 3, para 1, abortion 1, presented to the emergency department with a six-hour history of severe, persistent, dull pain in the right hypochondrium and 3 months of amenorrhea. She also complained of pain in her right shoulder and had experienced four episodes of non-projectile, non-bilious vomiting.

One month previously, she had attended an obstetrics and gynecology outpatient department with two months of amenorrhea and a positive urine human chorionic gonadotropin (HCG) test. However, neither transabdominal nor transvaginal obstetric ultrasound revealed signs of uterine or tubal pregnancy. She was scheduled for a follow-up ultrasound after two weeks but was lost to follow-up and presented again one month later as detailed above. Her past medical and surgical history were unremarkable.

On physical examination, the patient appeared acutely ill, exhibiting signs of distress due to pain, although her vital signs were stable except for a tachycardic heart rate of 106 beats per minute. Additionally, her conjunctivae appeared slightly pale. Her abdomen was slightly distended, with a visible lower uterine segment Pfannenstiel scar from a cesarean section. Diffuse tenderness was noted throughout the abdomen, with more pronounced tenderness, rebound tenderness, and guarding observed in the right hypochondrium. There was no shifting dullness or palpable mass. On a pelvic examination, the uterus was non-tender and normal in size, with no adnexal tenderness and no vaginal or cervical abnormalities were detected. No other significant findings were noted.

The laboratory workup revealed a white blood cell count (WBC) of 19,000/μL with a neutrophil percentage (NEU%) of 88.6, a red blood cell count (RBC) of 3.86 million/μL, a hemoglobin (HGB) level of 10.8 g/dL, a hematocrit (HCT) of 30.4 %, a platelet count (PLT) of 213,000/μL, and her blood group was AB negative. Additionally, renal and liver function tests (RFT and LFT) were within the normal range. A quantitative beta HCG test yielded a result of 168,100 mIU/mL.

**Fig. 1:**
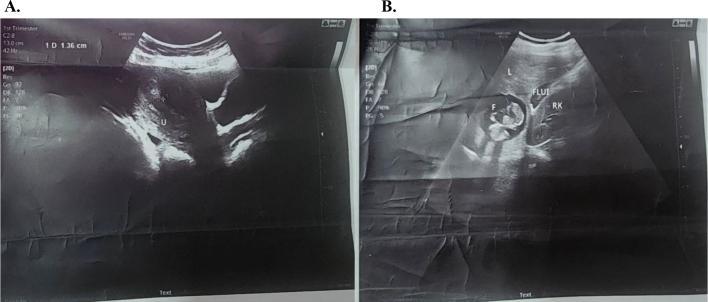
A. Pelvic ultrasound - sagittal plane– showing an empty uterus (labeled U) with a thin endometrium and minimal free posterior cul de sac fluid B. Abdominal ultrasound – longitudinal plane - showing liver (labeled L), fetus (labeled F) of crown-rump length measuring 5.75 cm, free fluid in the Morrison's pouch (labeled Flui) and right kidney (labeled RK).

On abdominopelvic ultrasound, the uterus still appeared normal in size, anteverted, with normal myometrial and cervical echo patterns. The endometrium showed normal thickness (1.2 cm), with no fluid, focal lesion, or mass observed in the endometrial cavity. Both ovaries appeared normal, with no ovarian cysts or adnexal masses identified. However, a pelvic collection with internal low-level echoes was noted. Ultrasound also revealed a normal-sized liver with a normal parenchymal echo pattern, sharp edges, and a smooth contour. A single, smoothly contoured gestational sac was observed in the right hepatic lobe, containing a fetus with demonstrable heart pulsations. The gestational age, based on crown-rump length (CRL), was measured at 5.75 cm, corresponding to 12 weeks and 0 days. Peritoneal fluid was also detected in the hepatorenal fossa, perisplenic space, interloop, and left paracolic gutter (see Fig. 1).

**Fig. 2:**
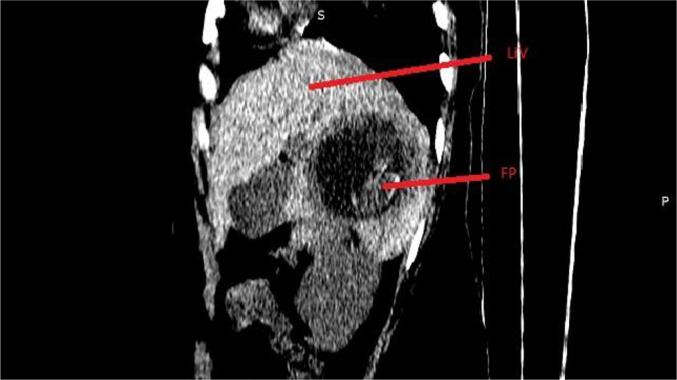
MPR image showing the location of the fetal pole (labeled FP) in the liver (labeled Li).

**Fig. 3:**
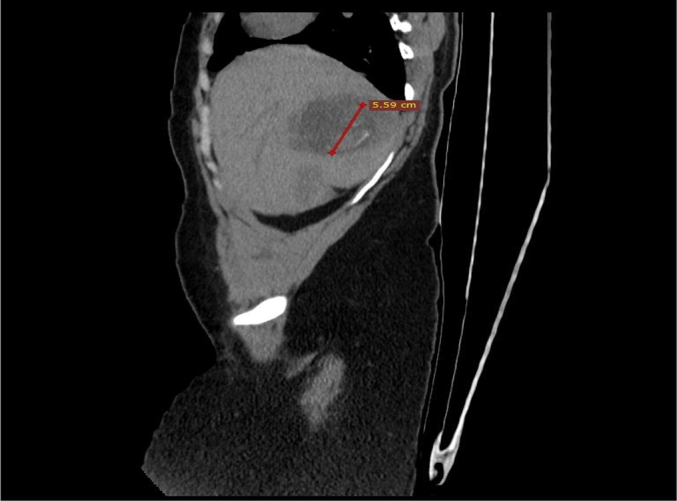
Sagittal image showing the fetal pole with internal calcific foci and CRL.

**Fig. 4:**
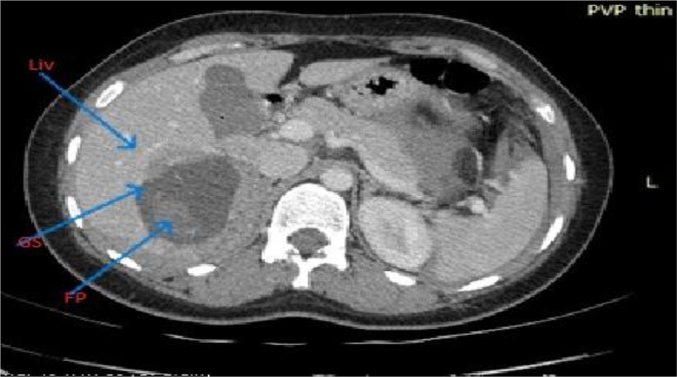
Axial post-contrast image showing (arrows) segment VI of the liver, gestational sac (GS), and fetal pole (FP) in segment VI of the liver (Liv).

**Fig. 5:**
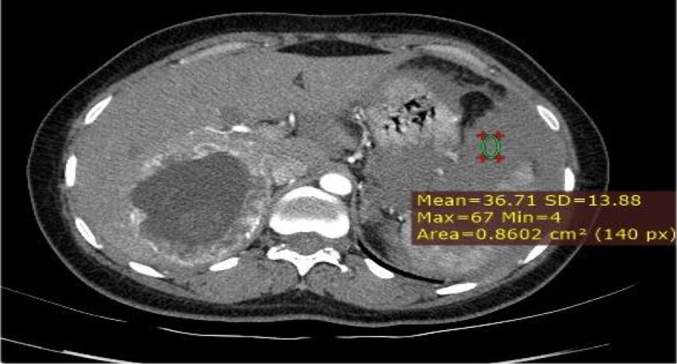
Hyperdense fluid collection in the perisplenic and pelvic peritoneal cavities.

**Fig. 6:**
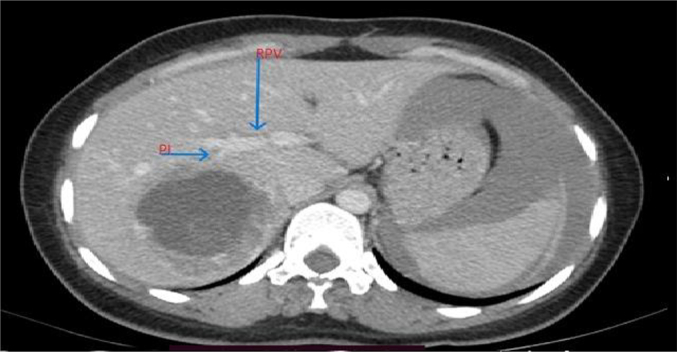
Gestational sac getting supply from a small posterior inferior segmental branch (PI) of the right portal vein (RPV).

**Fig. 7:**
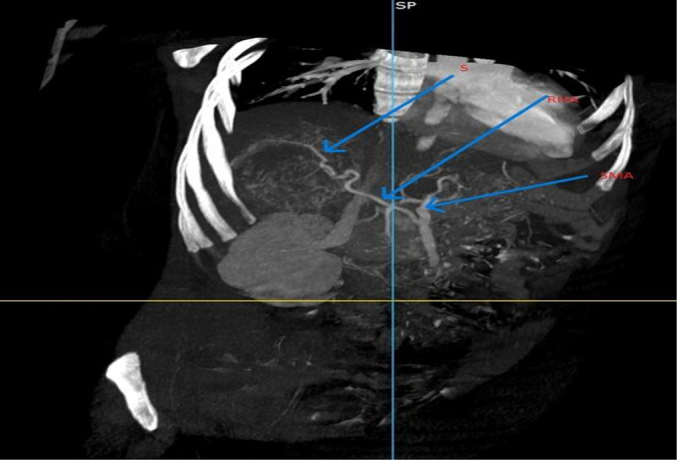
A variant hepatic arterial anatomy – right hepatic artery (RHA) (supplier (S) of the gestational sac) arising from the superior mesenteric artery (SMA).

Based on the patient's history, physical examination, laboratory results, and ultrasound findings, a provisional diagnosis of ruptured primary hepatic pregnancy was made. However, in light of her stable vital signs and the rarity of the case, a CT (computed tomography) scan with contrast was performed to ascertain the precise location and characteristics of implantation. A triphasic abdominal CT scan with contrast revealed a normal-sized liver with a smooth contour and homogeneous background parenchymal density. A 7.8 cm lesion was observed in the right lobe (segment VI) of the liver, characterized by heterogeneous density. The lesion exhibited peripheral hypodensity, with a predominantly cystic central area and a finely calcified hypodense focus. In the post-contrast study, the lesion demonstrated a hypervascular thick peripheral rim. An adjacent slightly hyperdense fluid collection was noted in the subcapsular region and hepatorenal recess. A large amount of slightly hyperdense free intraperitoneal fluid was observed in the perisplenic, paracolic, and pelvic peritoneal spaces. The uterus appeared to be grossly normal. The bilateral fallopian tubes and ovaries were normal (Fig. 2, Fig. 3, Fig. 4, Fig. 5, Fig. 6, Fig. 7).

After explaining the situation to the patient and obtaining informed consent, she was prepared for a laparotomy 8 h after the first presentation to the emergency department. Mifepristone 200 mg orally was administered, and 4 units of cross-matched blood were prepared. Surgery commenced under general anesthesia. An inverted L incision was made in the right upper quadrant, and upon entering the abdomen both clotted and non-clotted blood were found in the peritoneal cavity. The ruptured ectopic pregnancy was located in the posterior aspect of segment VI of the liver. Clotted blood was removed, and non-clotted blood was suctioned out. The right lobe of the liver was mobilized, and the Pringle maneuver was performed. The Pringle maneuver is a surgical technique that involves clamping the hepatoduodenal ligament to limit blood inflow to the liver by occluding the hepatic artery and portal vein. Upon mobilization of the liver, a well-formed fetus with an umbilical cord and placenta was expelled. We clamped the hepatoduodenal ligament while conducting a wedge segmental resection of the liver, specifically at segment VI, where the trophoblastic tissue was attached. Upon release of the vascular clamp, we observed minimal oozing from the liver surface. Initially, we attempted pressure packing to control this oozing. However, in areas where the raw surface continued to ooze, we applied Surgicel for additional hemostatic support, and a tube drain was left in the sub-hepatic space. The abdomen was washed out with normal saline and closed in layers. The blood loss was estimated to be 2000 mL, and the patient was transfused with 2 units of whole blood in the operating room.

The macroscopically normal fetus had a crown-rump length (CRL) of 5.5 cm (estimated gestational age [GA]: 14 weeks) and weighed 19.5 g. The cord was 8 cm long, and the placenta 7 × 5 cm, weighing 57 g, and was found to be friable. Biopsy samples were sent to the pathology department for further evaluation.

Following the operation, the patient was kept nil per os and administered analgesics and antibiotics. Additionally, she received 150 μg anti-D immunoglobulin and 50 mg/m2 intramuscular methotrexate. Her vital signs remained stable throughout the postoperative period. Approximately 200 mL of blood was drained via the tube, and she was transfused with an additional unit of whole blood.

**Fig. 8:**
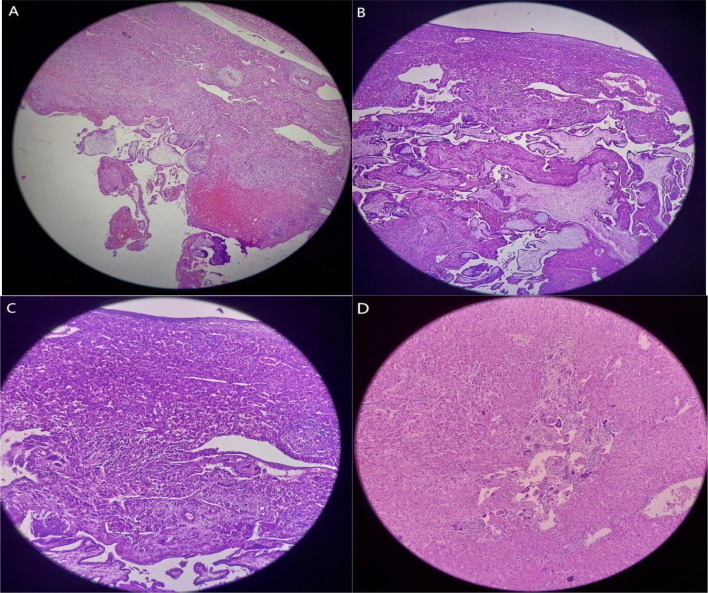
Biopsy images of the resected liver A. Chorionic villi invading a focally hemorrhagic hepatic tissue (Low-power view, H&E, 40×) B. Numerous chorionic villi infiltrating the hepatic tissue (H&E, 100×) C. Cytotrophoblasts and syncytiotrophoblasts infiltrating the hepatic tissue (H&E, 100×) D. Syncytiotrophoblasts in the hepatic tissue (H&E, 400×).

The biopsy sample grossly measured 7x5x4 cm, with a grey-brown partly circumscribed appearance and a rough surface, including attached friable tissue. Microscopic examination showed chorionic villi with cytotrophoblasts and syncytiotrophoblasts infiltrating into the hepatic tissue, along with focal periportal mononuclear inflammatory infiltrates and numerous foreign-body giant cells. Postoperative laboratory tests indicated elevated levels of AST (350.3 U/L) and ALT (405 U/L), with other parameters within normal ranges. The patient remained hospitalized for 2 days postoperatively with stable vital signs and was discharged with oral antibiotics and analgesics. Upon follow-up appointment scheduled a week after the surgery to assess her status her serum B-HCG level was 1674 Miu/mL. The serum HCG level returned to normal 3 weeks after the surgical management (see Fig. 8).

## 3 Discussion

Ectopic pregnancy is characterized by the implantation of a zygote at a site outside the endometrial cavity. The reported prevalence of ectopic pregnancies varies from 1 in 84 to 1 in 357 live births [4]. The fallopian tubes represent the most frequent site of extrauterine implantation, with abdominal pregnancies being uncommon, constituting 1.4 % of all ectopic pregnancies [5]. Pelvic localization is the prevailing site of implantation in abdominal pregnancies, with primary hepatic pregnancy representing an exceedingly rare subtype [6,7]. Among a cohort of 236 extrauterine pregnancies, merely eight instances of hepatic placental attachments were identified [4]. IN the literature, the youngest patient diagnosed with hepatic pregnancy was 18 years old, while the oldest was aged 46 years [8,9].

The diagnosis of primary abdominal pregnancy is made by Studdiford's criteria, which include the presence of normal bilateral tubes and ovaries with no evidence of recent or past pregnancy, no evidence of a uteroperitoneal fistula, and the presence of pregnancy-related exclusively to the peritoneal surface, early enough to eliminate the possibility of secondary implantation after primary tubal nidation [10,11]. The patient in the present case fulfilled this criterion. However, secondary abdominal pregnancy following the migration of the gestational sac into the abdominal cavity is more common than primary abdominal pregnancy [12].

Etiological factors associated with primary hepatic pregnancy include pelvic inflammatory disease (PID), a history of cesarean section, oral contraceptive use, intrauterine device use, and conception through assisted reproductive techniques [3,12]. While the present patient's history of cesarean section could theoretically have predisposed her to abdominal pregnancy, this typically leads to secondary abdominal pregnancy rather than primary. The proposed mechanism involves the possibility of migration through a microscopic uterine wall tear or via a uteroplacental fistula [13]. However, this scenario is improbable in this patient, given the absence of uterine abnormalities noted during preoperative imaging and operative exploration.

In cases of primary abdominal pregnancy, the zygote evades tubal capture and instead implants itself on the peritoneal surface or other highly vascularized organs, such as the liver, mirroring the circumstances observed in the patient's case. This phenomenon is hypothesized to occur as the zygote navigates the circulatory pathway of peritoneal fluid, facilitated by peristaltic and respiratory movements. Consequently, the zygote ascends through the paracolic gutters, eventually reaching the right upper abdomen. Within this region, the inferior surface of the right lobe emerges as the most probable site of implantation, owing to its lowest position in the supine posture and increased vascularity [[14], [15], [16]].

A subgroup of patients initially present with slow-onset, nonspecific gastrointestinal or hepatobiliary symptoms. Common complaints include epigastric pain, nausea, and vomiting, which can easily be mistaken for other conditions such as cholecystitis, hepatitis, dyspepsia, and peptic ulcer disease, leading to challenges in making the initial diagnosis and causing delays in treatment [2,17]. This symptomatology may arise due to the proximity of the most frequent site of pregnancy attachment, the inferior surface of the right lobe of the liver, to the gallbladder and duodenum [16].

More specific symptoms may arise due to slow intra-abdominal hemorrhage, eventually manifesting as signs suggestive of a ruptured ectopic pregnancy. Indeed, this is how most patients initially present [7,18]. This scenario was observed in the patient's case. However, the onset of symptoms can be ambiguous, and provisional diagnoses may be misleading. Given that blood loss can occur gradually and hemodilution can maintain normal blood pressure, healthcare professionals may initially overlook the possibility of a ruptured ectopic pregnancy. Nevertheless, this can progress to severe anemia and hemorrhagic shock. Therefore, in cases where women of reproductive age present with atypical abdominal symptoms, a high level of suspicion, coupled with a timely radiologic investigation, is imperative to avoid potentially life-threatening intra-abdominal bleeding from an abdominal ectopic pregnancy and to enhance favorable clinical outcomes [12]. This is particularly pertinent for patients with gradually deteriorating vital signs indicative of slow-onset hypovolemic shock. Healthcare providers should not solely rely on typical features such as pelvic pain, amenorrhea, and vaginal bleeding when considering the diagnosis of ectopic pregnancy [12].

The potential mortality rate associated with hepatic pregnancy is reported to be five to seven times higher than that observed in other types of ectopic pregnancies [3]. Diagnostic delay elevates the risk of complications such as hemorrhage into the abdominal cavity [12,19]. Early abdominopelvic ultrasonography is instrumental in expediting diagnosis and mitigating delays caused by ambiguous gastrointestinal symptoms [5]. Transabdominal ultrasound confirms the location and aids in estimating gestational age. In early pregnancy, the gestational sac presents as a cystic lesion containing the embryonic pole-yolk sac complex, with a variable presence of cardiac activity. Detection of a surrounding peri-gestational hematoma serves as an early indicator of an impending rupture. Employing color Doppler can be advantageous in delineating the blood supply [12].

During the later stages of pregnancy, the fetus and placenta are discernible within the abdominal cavity, distinct from the uterus. Transvaginal ultrasonography (USG) is a pivotal tool for confirming the absence of an intrauterine pregnancy and checking the fallopian tubes. However, it is important to recognize that limiting imaging solely to reproductive organs risks can miss abdominal pregnancy.

In clinically stable patients, additional imaging techniques such as CT scans and MRI play a vital role in precisely defining regional anatomy and facilitating perioperative planning by identifying the placental implantation site and aiding in the decision-making process regarding whether to retain or remove the placenta during laparotomy [4,7,15,20]. Furthermore, given the potential for confusion with other conditions due to the unusual location of the pregnancy, such as being mistaken for a liver tumor, these imaging modalities serve to exclude such differential diagnoses [3,21,22].

When considering whether to utilize a CT scan or an MRI, it is important to note that the superior soft-tissue resolution of MRI allows for precise identification of extrauterine gestational sacs, adnexal masses, and hemoperitoneum. This level of detail can offer crucial information to help make clinical and surgical management decisions. However, it is vital to use MRI only when patients are stable and when additional diagnostic information is needed to guide patient care [23]. On the other hand, contrast-enhanced computed tomography (CECT) serves as an urgent diagnostic imaging modality. It is proficient in identifying the site of bleeding and surveying the abdominal cavity. CECT exhibits high sensitivity in predicting ectopic implantation sites. CECT may be particularly beneficial in scenarios where MRI is unavailable, making it a viable option for the diagnosis of ectopic pregnancy in acute settings [24].

Management options documented in the literature span from conservative approaches to surgical interventions, encompassing both laparoscopic and laparotomy. Conservative options entail the administration of methotrexate in patients with a Fernandez score favoring medical management, particularly when the patient remains hemodynamically stable [17,25]. An alternative approach for clinically stable patients involves ultrasound-guided feticide, typically achieved via intrathoracic potassium chloride injection, followed by methotrexate administration to the mother [26,27].

Resection of hepatic pregnancy via surgical means can be achieved through either laparoscopy or laparotomy, with the decision contingent upon the risk of hemorrhage [5,15,28]. Open surgery is the preferred approach for patients presenting with hemoperitoneum and unstable vital signs [2,29]. Priority is placed on mitigating or preventing hemorrhage, with various surgical procedures employed for this, including hepatic artery ligation, omental transplantation, wedge resection or lobectomy, liver packing, and leaving the placenta in situ [12,15].

These surgical management strategies can be complemented by additional interventions. Preoperative catheter embolization of the arterial feeder is utilized to reduce the risk of bleeding, especially in cases where there is deep implantation into the hepatic parenchyma, resulting in only partial pregnancy resection [30]. While postoperative hepatic artery embolization was attempted in one report, its efficacy in controlling hemorrhage has been limited, with the authors suggesting that preoperative arterial embolization may yield better outcomes in retrospect [5]. Another option involves postoperative methotrexate administration, particularly in cases where the placenta is left in situ or there is a rise in serum human chorionic gonadotropin (HCG) levels despite resection, or intraoperative injection of methotrexate directly into the sac in cases of unresectable pregnancy [5,9,31,32]. Methotrexate facilitates the expedited degeneration of residual trophoblastic tissue. Additionally, preoperative administration of mifepristone is employed in certain cases, as it has demonstrated improved cure rates when combined with methotrexate in ectopic pregnancies [33]. Mifepristone acts by reducing vascularity and inducing degeneration and necrosis of villi [34]. This action contributed to less intraoperative bleeding and better surgical outcome in this case. Preoperative mifepristone and postoperative methotrexate as well as surgical resection were used. This approach was taken to minimize the risk of post-surgical trophoblastic remnants.

## 4 Conclusion

The present case of primary hepatic pregnancy underscores the importance of maintaining a high index of suspicion for ectopic pregnancies, even in rare locations, when managing patients with atypical abdominal symptoms. Timely diagnosis and intervention are paramount, as the potential for severe intraperitoneal hemorrhage significantly increases maternal morbidity and mortality. Therefore, a proactive approach that prioritizes early recognition and management is vital to improving outcomes in these high-risk scenarios.

The following is the supplementary data related to this article.

Supplementary video 1 An abdominal ultrasound shows a fetus within the liver with visible heart pulsations. Supplementary video 1
